# Supplementary material for: Time-to-Death approach in revealing Chronicity and Severity of COVID-19 across the World
Source: PLoS One. 2020 May 12;15(5):e0233074. doi: 10.1371/journal.pone.0233074 (PMC7217458; doi:10.1371/journal.pone.0233074)
Supplement: S1 Appendix — (DOCX) [file pone.0233074.s001.docx]

**Appendix**

Suppose $T$denotes the duration of exposure, since the COVID-19 virus infected cases were registered in a country. Let $N_{i}$denotes the number of cumulative cases registered on i*^th^* day of the country, and $D_{i}$denotes the number of cumulative deaths reported among $N_{i}$infected cases, where $i=1,2,.., T.$ Let, $\pi_{i}$ denotes the mortality rate due to COVID-19 virus on *j^th^* day in country. The method of generalized linear modeling has used to predict the properties of duration-deaths relationship, and the mortality rate due to COVID-19 is obtained as

$$\Phi^{-1}\left( \pi_{i} \right)=\left( \beta_{0}+\beta_{1}T \right) \ldots.(1)$$

$$\Rightarrow\pi_{i}=P\left( Death due to Covid 19 \right)=\Phi\left( \beta_{0}+\beta_{1}T \right) \ldots..(2)$$

where$\Phi$ is the cumulative distribution function used to model the probability (using Gompertz distribution), where $\beta_{0}$ is the overall intercept and $\beta_{1}$ is the common slope on duration (T).For the present study, Gompertz distribution has used as $\Phi$ to estimate the probability$\pi_{i}$ of country, and the model has used for linking the duration of exposure to the probability of death though the chosen $\Phi$. Once the parameters, viz., $\beta_{0}$ and$\beta_{1}$, are estimated, the predicted death probability of the country, $\hat{\pi}_{i}$ is calculated by following the Binomial model as

$$Death\left( D_{ij} \right)\sim Binomial \left[ N_{ij}, \Phi(\beta_{0}+\beta_{1}T_{i}) \right]. \ldots\ldots(3)$$

As the quantity interest is the duration (in terms of days) of exposure, which has a specific probability of death among COVID-19 infected individuals; therefore, this duration is known as lethal duration, ${LD}_{\pi}$, for a given probability of death occurrence ($\pi$) and is estimated using equation (2) as

$$\Phi^{-1}\left( \pi_{i} \right)=\beta_{0}+\beta_{1}{LD}_{\pi} \ldots\ldots(4)$$

$$\hat{LD}_{\pi}= \frac{\Phi^{-1}\left( \pi_{i} \right)-\hat{\beta}_{0}}{\hat{\beta}_{1}}\boldsymbol{\ldots\ldots(5)}$$

and its 95% confidence interval at level of significance $(\alpha)$ is obtained as

$$\boldsymbol{\Phi}^{\mathbf{-1}}\left( \boldsymbol{\pi}_{\boldsymbol{i}} \right)\boldsymbol{\pm}\boldsymbol{z}_{\boldsymbol{\alpha/2}}\sqrt{Var\boldsymbol{(}\boldsymbol{\Phi}^{\mathbf{-1}}\left( \boldsymbol{\pi}_{\boldsymbol{i}} \right)\boldsymbol{)}}$$
