# Supplementary material for: Time-to-Death approach in revealing Chronicity and Severity of COVID-19 across the World
Source: PLoS One. 2020 May 12;15(5):e0233074. doi: 10.1371/journal.pone.0233074 (PMC7217458; doi:10.1371/journal.pone.0233074)
Supplement: Attachment — Submitted filename: Response to Reviewers_Covid 19.docx [file pone.0233074.s002.docx]

**RESPONSE TO REVIEWERS’REPORT(S)**

| **Reviewer 2** | **Response** |
| --- | --- |
| (a) what are the underlying assumption/s of this modelling technique; please address them (if any) clearly in the method section of the paper; | The suggestion has discussed in 6^th^ line of para 2 of page 6 of Section 2. |
| (b) across the globe the number of COVID-19 cases are dropping due to social distancing and other measures. My concern is whether the model can be useful to capture this downfall of confirmed cases. | The aspect of social distancing and other measures are taken care while defining the 95% confidence interval for the lethal duration. The suggestion has discussed in 13^th^ line of para 1 of page 7 of Section 4. |
| **Reviewer 3** | **Response** |
| Objective: It should be specific. • The author mixed justification of the study and possible recommendation of the study under objective, e.g. first 12 sentences under the headline (Objective) belong to justification.  • And part of last sentence e.g. “Which will help in planning strategies required to be implemented in time”. It appears as a recommendation based on study findings. | The suggestion has incorporated in third and first and second paras of page 5 in Section 1 of the revised manuscript.  The suggestion has incorporated in the revised manuscript at 5^th^ line of fourth para in page 8 of Section 5. |
| Methodology: • Based on mortality rate the author sets the affected countries into three category. As per categories, the predicted mortality rates were grouped into 3 groups. Group 1: 1 to 5%, Group 2: 6 to 10% and group 3: 20 to 50%. It is not clear to me what about 11 to 19% is. | To address the change in mortality rates, the classes were defined with either 1% or 10% increment in mortality rates, which has been incorporated in first para of page 6 in Section 2 of the revised manuscript. |
| • Methodology means what the author does to conduct the study. Therefore, first two sentences in page 6 may be reformulated. | An appropriate changehas been incorporated in second para of page 6 in Section 2 of the revised manuscript. |
| Result: • 7th line Page 6, it will be 28th. | Done in the revised manuscript. |
| • Group 3: 20 to 50%. Percentage of 11 to 19 is missing. | To address the change in mortality rates, the classes were defined with either 1% or 10% increment in mortality rates, which has been incorporated in first para of page 6 in Section 2 of the revised manuscript. |
| • 11th line “For category I country, the prediction was…………………. having mortality rates of 20-50%.” It is same as methodology. Sentences needs to be modified if it is result | Done in the revised manuscript. |
| I am not a statistician. Therefore, a statistician must critically analysis statistical part e.g.:  •Statistical formula  •Figure on inverse probability analysis for total deaths  • Pattern of probability death occurrence | Both Reviewers 1 and 2 found the statistical analysis has been performed appropriately and rigorously. |
| Discussion: • Discussion part may be shorten by omitting duplication of sentences used in result section. | An appropriate change has been incorporated to the first para of page 7-8 at Section 4 of Discussion in the revised manuscript. |
| • There is no reference in discussion. If similar study on prediction of lethal duration is conducted on other communicable diseases that may be used as reference for discussion. | Reference no [12-13] has been updated at first para of page 7 at Section 4 in the revised manuscript, which is related with forecasting and application of the method to antimicrobial agent’s activities. But no such work is found that is related with the prediction of lethal duration in connection with any communicable diseases. |
| Conclusions: • Contents of conclusions should include only finding of the study. In one sentence future term is used that needs to be corrected. | Done in the revised manuscript. |
| • First sentence and third sentences of conclusions may be deleted. | Done in the revised manuscript. |
| • Part of last sentences e.g. “Would eventually help to implement strategies to tackle this pandemic by taking necessary appropriate steps.” Better to mention what are the necessary steps; if the author identified some of the necessary steps. | Section 5 of Conclusion has been revised. |

**Note: All of the changes in the revised manuscript are highlighted using YELLOWcolour.**
